# C8 medium chain triglycerides benefit energy metabolism and modulate inflammatory lipids in cats with chronic kidney disease

**DOI:** 10.1093/jas/skag193

**Published:** 2026-09-02

**Authors:** Brielle N Gordon, Kiran S Panickar, Regina L Hollar, Selena K Tavener, Dennis E Jewell, Matthew I Jackson

**Affiliations:** Science and Technology Center, Hill’s Pet Nutrition, Inc, Topeka, KS 66617, United States; Science and Technology Center, Hill’s Pet Nutrition, Inc, Topeka, KS 66617, United States; Science and Technology Center, Hill’s Pet Nutrition, Inc, Topeka, KS 66617, United States; Science and Technology Center, Hill’s Pet Nutrition, Inc, Topeka, KS 66617, United States; Department of Grain and Food Science, Kansas State University, Manhattan, KS 66506, United States; Science and Technology Center, Hill’s Pet Nutrition, Inc, Topeka, KS 66617, United States

**Keywords:** beta-hydroxybutyrate, feline metabolism, inflammation, renal disease

## Abstract

Chronic kidney disease (CKD) is characterized by inflammation and irreversible damage to the structure and function of kidneys. The kidneys require large amounts of energy to maintain their structural integrity and function, and in CKD there is a disruption of renal energy metabolism due to decreased oxidative phosphorylation and mitochondrial function. The canonical medium-chain fatty acids (MCFAs) are caproic (C6:0), caprylic (C8:0), and capric (C10:0) acids, while lauric acid (C12:0) is considered an intermediate fatty acid. The purpose of this study was to assess circulating markers of energy status and inflammation in felines with CKD when fed trioctanoin (C8 MCT), a medium-chain triglyceride (MCT), in the presence of fish oil. The C8 MCTs are hydrolyzed into MCFAs, which are more efficiently digested, transported to the liver, and absorbed into mitochondria than intermediate or long chain fatty acids. They also generate higher levels of ketone bodies such as beta-hydroxybutyrate (BHB), which is a preferred source of energy for kidneys. Various lipid metabolites and BHB contribute to energy metabolism and modulate inflammation. The subjects were cats with naturally occurring CKD (at least International Renal Interest Society (IRIS) stage one with symmetric dimethylarginine (SDMA) ≥14.0 μg/dL for at least 3 mo; *n* = 16) housed at Hill’s Pet Nutrition Center. In a cross-over study, based on sex and age cats were assigned to the order of feeding of a control food (dry food for management of renal disease) and test food. On an as-fed basis, pork fat was decreased from 12.3% in the control food to 4.3% in the test food, allowing for the addition of 8% C8 MCTs. Fasting blood and urine were collected after feeding each food for 8 wk. Analyses included assessing plasma metabolomics, serum and urine biochemistries, and complete blood counts. Fasting levels of circulating BHB were increased by the test food and carnitine adducts showed a bidirectional effect, as compared to control food (*P* < 0.05). There were also decreases in inflammatory lipid mediators including dihydro- and hexosylceramides, lysophospholipids, and phosphatidylcholines with test food (*P* < 0.05 vs control), suggesting reduced inflammatory signals. These results indicate feeding C8 MCTs to cats with CKD increases available energy from ketones and decreases lipids involved in inflammation, supporting a beneficial role of C8 MCTs in the nutritional management of CKD in cats.

## Teaser

Feeding C8 MCTs to cats with CKD enhances energy availability through ketones and reduces inflammation-associated lipids. Consequently, C8 MCTs can aid in managing CKD in cats by providing more accessible energy and reducing inflammation.

## Introduction

Renal disorders are a leading cause of death in cats and affect 42%–80% of cats greater than 10 yr old (Marino et al. 2014). Renal disease is accompanied by poor energy status and systemic inflammation. The kidneys continuously consume large amounts of energy to maintain their function and structural integrity (Houten et al. 2016; Cuenoud et al. 2020; Jang et al. 2020; Panov et al. 2022). Disruption of energy metabolism in the kidney, including downregulation of genes related to fatty acid beta-oxidation (FAO) and oxidative phosphorylation in the mitochondria, has been shown to contribute to chronic kidney disease (CKD) in a longitudinal mouse study (Harzandi et al. 2021).

The canonical medium-chain fatty acids (MCFAs) include caproic acid (C6:0), caprylic acid (C8:0), and capric acid (C10:0), while lauric acid (C12:0) is considered to be an intermediate or transitional fatty acid as it does not exhibit the same biochemical properties as the canonical MCFAs. Dietary fatty acids are typically consumed in the form of triglycerides, and hydrolysis of medium-chain triglycerides (MCTs) to MCFAs is more efficient than hydrolysis of intermediate or long chain triglycerides to intermediate or long chain fatty acids (Bach et al. 1996; Birk and Brannon 2004; Marten et al. 2006; Fontbonne et al. 2011). Additionally, MCFAs are directly transported to the liver via the portal vein (Trevizan et al. 2010) and do not require membrane transporters for cellular uptake or carnitine palmitoyl transferase 1 (CPT1) for transport into the mitochondria (Roopashree et al. 2021). They also generate higher levels of ketone bodies (see Li et al. 2025 for review). Beta-hydroxybutyrate (BHB) is an energy dense molecule that not only serves as an efficient fuel but also exerts anti-inflammatory, antifibrotic, and antioxidative effects as recently reviewed in the context of human kidney disease (Athinarayanan et al. 2024). Evidence from human studies indicates that BHB is widely utilized across the body as a substrate for adenosine triphosphate (ATP) production and appears to be the preferred energy source for the kidneys (Cuenoud et al. 2020). At the mechanistic level, BHB functions as a histone deacetylase inhibitor (Shimazu et al. 2013), which leads to inhibition of Nod-like receptor family pyrin domain containing 3 (NLRP3) signaling and thereby reduces inflammation (Youm et al. 2015). In a diabetic rat model, BHB has also been reported to upregulate Matrix metallopeptidase 2, which in turn helps attenuate glomerulosclerosis (Luo et al. 2020).

MCTs have been shown to suppress the inflammatory response and increase levels of anti-inflammatory factors in various animal models (Carlson et al. 2015; Lee and Kang 2017; Yu et al. 2019; Gaete et al. 2023). Inflammation is a normal and vital component of various biological processes and requires tightly regulated pro-inflammatory and anti-inflammatory responses to protect the host and maintain homeostasis. Improper regulation of inflammation can lead to increased production of pro-inflammatory cytokines, oxidative stress, fibrosis, tissue damage, reduced organ function, and eventual organ failure (see Brennan et al. 2021 for review). Thus, dysregulated, systemic inflammation plays a major role in the progression of CKD and its related complications (see Rapa et al. 2019 for review). A recent review of human CKD conveys factors including dyslipidemia, metabolic syndrome, oxidative stress, and compromised gut health contribute to the systemic inflammation that affects the development, maintenance, and progression of CKD by promoting renal fibrosis and injury (Kadatane et al. 2023). Furthermore, CKD can cause or exacerbate conditions such as cardiovascular disease, atherosclerosis, diabetes, anemia, and renal cell carcinoma (Kadatane et al. 2023). Numerous human and mouse models have shown various lipids including polyunsaturated fatty acids (PUFAs), ceramides, and sphingosines to have immune modulating properties (Huwiler and Pfeilschifter 2009; Huang et al. 2010). Data from a recent study in healthy cats indicated that a food containing fish oil and enriched with a mixture of C8 and C10 MCTs (CAPTEX-355, 51.4% caprylate (C8:0), 39.1% caprate (C10:0); ViaChem Inc, Plano, TX, USA) may confer renal protection (Jackson and Jewell 2020).

Investigations into MCTs have been multifarious and indicate promise for dietary interventions. Nonetheless, the benefits of dietary MCTs in cats with CKD remain unstudied. Given the observed effects of MCTs on energy metabolism and inflammation, we investigated the impact of dietary C8 MCTs in cats with CKD.

## Materials and methods

### Ethics statement

This study was approved by the Institutional Animal Care and Use Committee, Hill’s Pet Nutrition, Inc., Topeka, KS (Protocol Number: FP927.1.1.0-A-F-D-ADH-MULTI-133-KID) and the Animal Welfare Committee at Hill’s Pet Nutrition. All protocols in this study complied with the National Institutes of Health guide (National Research Council 2011), and no invasive procedures were utilized.

### Animal care and management

This study was conducted at the Hill’s Pet Nutrition Center, where cats were housed in individual enclosures that offered 14 square feet of floor space and measured 98 inches in height. These enclosures included five vertical/climbing levels and provided access to natural light. Cats received daily human interaction for approximately 1 hr and spent up to 7 hr daily in a group environment outside their enclosures, which included access to sun porches. Enrichment was provided through daily attention from animal care technicians, as well as a variety of rotating toys (mice, balls, kickaroos, rotated every 2 wk). Scratchers, fluffy bedding, cat wheels, cat TV, boxes, paper bags, grooming, and supervised laser toys or walks/stroller rides were also available.

### Food formulation and production

Two dry extruded foods for management of feline renal disease with the same base formula chiefly composed of brown rice, corn gluten meal, fresh chicken, whole wheat, and barley, in order of preponderance, were tested. Both foods were formulated to contain the same level of fish oil and met the nutritional requirements established by the Association of American Feed Control Officials for maintenance of adult cats. On an as-fed basis, the control food contained 12.3% pork fat, which was reduced to 4.3% in the test food to allow for addition of 8% C8 MCTs. The source of C8 MCTs was CAPTEX 8000 (ABITEC Corporation, Janesville, WI, USA), which had a chain length distribution of 0% caproic acid (C6), 99.6% caprylic acid (C8), 0.4% capric acid (C10), and 0% lauric acid (C12). The analytical nutrients of the control and test foods are shown in Table S1.

### Study design and measurements

Sixteen domestic shorthair cats with naturally occurring CKD were identified, all housed at the Hill’s Pet Nutrition Center, Topeka, KS. CKD was defined as having an International Renal Interest Society (IRIS) stage of at least 1 (International Renal Interest Society 2023) with symmetric dimethylarginine (SDMA), a biomarker of kidney dysfunction, ≥14.0 µg/dL for at least 3 mo before the start of the study (Table S2). The cats were assigned to group 1 or group 2 based on sex, age, and body weight (Table 1). Each group consisted of four neutered males and four spayed females. Immediately before the start of the treatment phase, the mean age and body weight of group 1 was 11.3 yr and 5.1 kg, respectively, while group 2 was 10.9 yr and 5.0 kg, respectively. When the treatment phase started, four cats were classified as IRIS stage 1, eleven cats were IRIS stage 2, and one cat was IRIS stage 3.

**Table 1:** Animal signalment and intakes.

	Group 1			Group 2			LMM, P		
	**Prefeed**	**Control**	**Test**	**Prefeed**	**Control**	**Test**	**Tx Group**	**Food**	**Tx Group** × **Food**
**Age, year**	11.3 ± 1.0			10.9 ± 0.8					
**Body weight, kg**	5.1 ± 0.2	5.1 ± 0.2	5.0 ± 0.2	5.0 ± 0.4	4.7 ± 0.4	4.8 ± 0.4	0.437	0.969	0.084
**Neutered male, *n***	4			4					
**Spayed female, *n***	4			4					
**IRIS 1**	3			1					
**IRIS 2**	4			7					
**IRIS 3**	1			0					
**Intake, cal/MBW**	59.1 ± 2.9	60.6 ± 2.3	65.2 ± 3.9	72.0 ± 7.2	78.4 ± 10.7	73.4 ± 7.1	0.064	0.945	0.108

Data are mean ± standard error or *n*.

IRIS, International Renal Interest Society; cal/MBW, calories per metabolic body weight; Tx, treatment.

Before the experimental phases, cats underwent a 7-d washout period during which all cats were fed the control food (Figure 1). The cats then entered a cross-over study consisting of two consecutive 8-wk experimental feeding phases with no washout period in between, as each 8‑wk phase was considered sufficient to wash out the effects of the preceding food and induce the effects of the next food. Group 1 received the control food in the first phase followed by the test food, whereas group 2 received the test food first followed by the control food. Fresh water was available *ad libitum* and the amount of food calculated to maintain a healthy body weight was provided in individually housed food bowls daily. Feed intakes were recorded daily and body weights weekly for the duration of the study. Feed intakes and body weights exhibit neither a food effect nor a treatment group effect by univariate linear mixed model (LMM) throughout the treatment phase (Table 1). One cat in group 2, who started the study at IRIS stage 2, did not complete the trial due to a rapid decline in renal function. It was concluded to be a nontreatment-related health issue as determined by a veterinarian.

**Figure 1:**
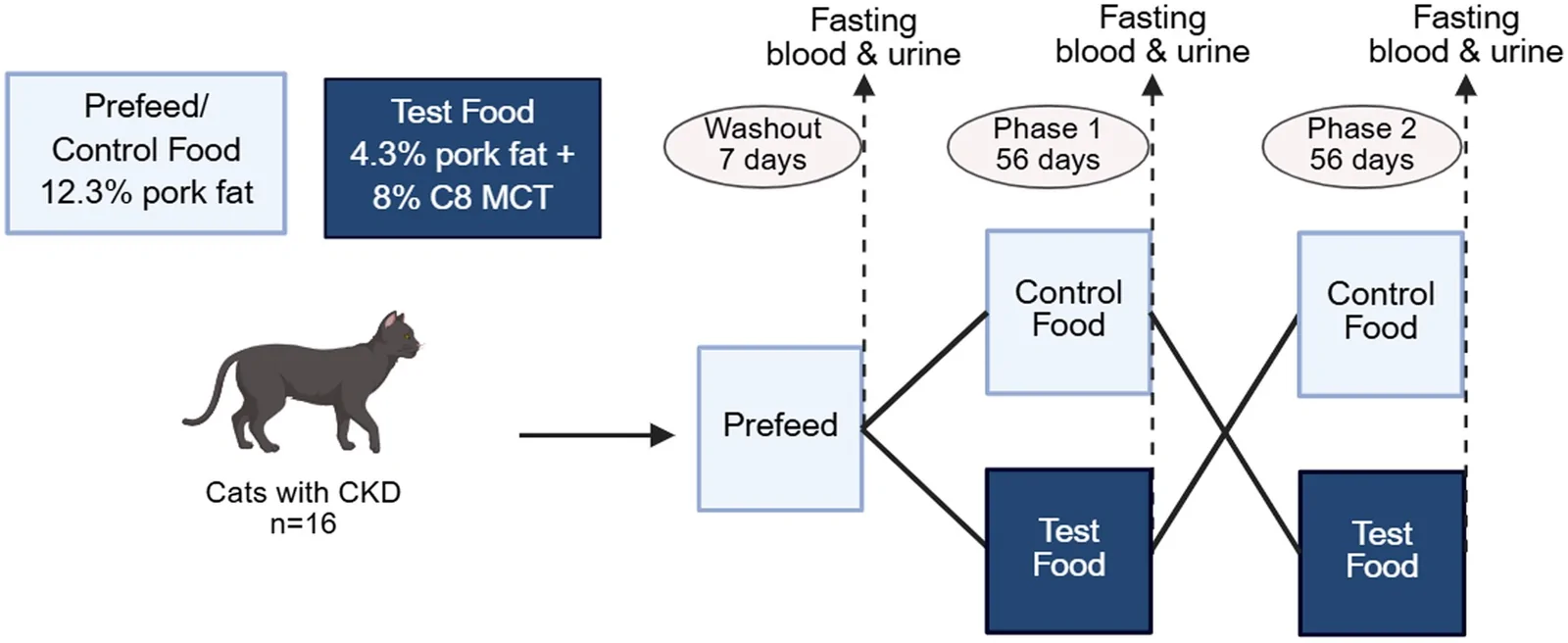
Cross-over study design. After a 7-d washout on control food (prefeed), cats completed two consecutive 8-wk feeding phases with no intermediate washout. Group 1 received control food followed by test food; Group 2 received test food followed by control food. Fasting blood and urine samples were collected at the end of the washout period and each experimental feeding phase. CKD, chronic kidney disease; MCT, medium chain triglyceride.

At the end of each experimental feeding period cats were fasted for approximately 16 hr and blood and urine samples were collected. Clinical serum (Cobas 6000 series, Roche Diagnostics, Indianapolis, IN, USA) and urine biochemistries and complete blood counts (CBCs; XT-2000 v Automated Hematology Analyzer, Sysmex America Inc, Lincolnshire, IL, USA) were performed immediately after collection by an in-house laboratory. These clinical instruments are calibrated daily to avoid batch effects. Urine biochemistries were analyzed as previously described (Anthony et al. 2023). Briefly, urine total protein and creatinine were measured on the Cobas instrument listed above; urine pH was measured with a potentiometric pH meter; urine specific gravity (uSG) was measured with a refractometer; the minerals calcium, magnesium, phosphorus, potassium, and sodium were analyzed by inductively coupled plasma-optical emission spectroscopy; sulfate, chloride, and citrate were analyzed by ion exchange chromatography; oxalate was analyzed by ion exclusion chromatography; ammonium was detected by a reaction that produces indophenol blue, which was then measured by spectrophotometry; these measurements were then used to calculate relative supersaturation using the EQUIL-HL21 program. Analysis of SDMA was completed by a commercial laboratory (IDEXX Inc, North Grafton, MA, USA) and analysis of plasma metabolomic profiles was performed by Metabolon (Morrisville, NC, USA) as previously described (Hall et al. 2018). Blood samples collected for analysis at external laboratories were placed immediately on ice, aliquoted, and subsequently frozen at −80°C within 2–3 hr of collection, and were kept at −80°C until shipment on dry ice to their respective laboratories. All samples were shipped simultaneously to avoid batch effects.

### Statistical analysis

Statistical analyses were performed in JMP Pro, version 18 (SAS Institute, Cary, NC, USA) using a LMM analysis, with food being the fixed effect and animal being the random effect, and multivariate analysis of variance (MANOVA) using the Identity function, fitting an individual model for each metabolite before performing a joint test. Clinical analytes showed no statistical effects of diet order, indicating the absence of a carryover effect, and metabolomics data were processed and analyzed using standard bioinformatics pipelines appropriate for large-scale datasets. Significance was set at *P* < 0.05 and false discovery rate was not performed as this was an exploratory pilot study. Statistics were performed on untransformed data for all clinical results. Metabolomics data were log2 transformed and analysis was performed on the transformed values.

## Results

### Impact of dietary MCTs on clinical blood chemistry, complete blood count, and urinalysis

The mean SDMA of all cats immediately before the start of the treatment phase was slightly above the normal reference range (0–14 µg/dL), and was maintained at this level throughout the treatment phase (Table S3). The mean immature reticulocyte fraction (IRF) of all cats immediately before the start of the treatment phase was well above the normal reference range and, although it was reduced by the test food, remained above the reference range (Table S4). The mean reticulocytes% of all cats immediately before the start of the treatment phase was below the normal reference range, and was maintained at this level throughout the treatment phase.

Consumption of the C8 MCT-enriched food increased circulating BHB levels in the blood (% difference = 22.4; *P* = 0.008; Figure 2) by univariate LMM. An increase in BHB was confirmed by a second method, the semi-quantitative untargeted plasma metabolomics analysis (Log2 fold change (FC)  = 0.32; *P* = 0.020). Treatment effects were also shown for clinical blood chemistry results including an increase in albumin/globulin (Alb/Glob) ratio (% difference = 7.3; *P* = 0.046) and decreases in cholesterol (% difference = −24.4; *P* = 1.2 × 10^−5^), alkaline phosphatase (ALP; % difference = −11.1; *P* = 0.018), and globulin (% difference = −3.4; *P* = 0.029). Complete blood count results exhibited a treatment effect for IRF (% difference = −12.0; *P* = 0.002) and red blood cell distribution width (RDW; % difference = −0.4; *P* = 0.044), with both being decreased. Urine total protein (% difference = −19.7; *P* = 0.028) and creatinine (% difference = −8.3; *P* = 0.038) levels also exhibited a treatment effect, with both being decreased (Table S5).

**Figure 2:**
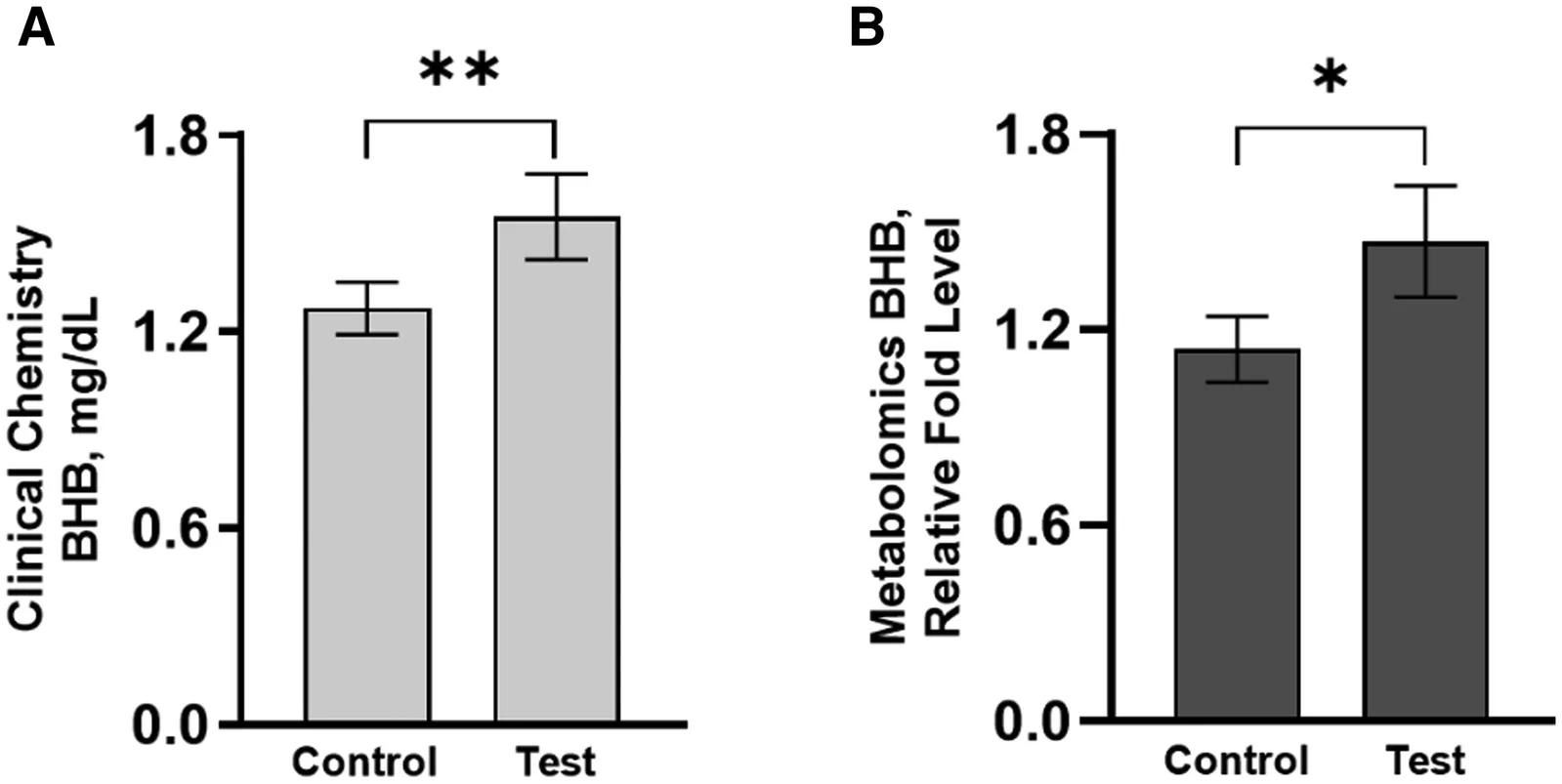
BHB levels as assessed by two complimentary methods. BHB levels showed similar results when measured by A) clinical chemistry and B) semi-quantitative untargeted plasma metabolomics. A) Data are mean ± standard error. B) Data are mean ± standard error of relative fold level. *P < 0.05 compared with control food. **P < 0.01 compared with control food. BHB, beta-hydroxybutyrate.

### Impact of dietary MCTs on the global plasma metabolome

Plasma metabolomics analysis identified 801 total metabolites, with 222 (27.7%) of these being different between control and test foods by univariate LMM (Table 2). Out of the 222 metabolites which showed a treatment effect, 75 (33.8%) were increased and 147 (66.2%) were decreased. The affected metabolite classes included amino acids (9.8% increased, 7.3% decreased), carbohydrates (4.3% increased, 8.7% decreased), cofactors and vitamins (13.3% increased, 16.7% decreased), energy (0.0% increased, 11.1% decreased), lipids (10.0% increased, 31.7% decreased), nucleotides (8.3% increased, 6.3% decreased), peptides (13.8% increased, 6.9% decreased), and xenobiotics (6.6% increased, 3.3% decreased). The dietary intervention evaluated in this study consisted of lipids provided as C8 triglycerides, and MCTs are known to modulate both lipid-derived energy pathways and lipid-mediated immune processes. Accordingly, our analyses were focused on metabolites involved in energy provision and lipid-mediated immune modulation. In addition, a small number of individual metabolites falling outside these predefined classes, but considered biologically relevant to this study, were also examined in greater detail.

**Table 2:** Summary of detected and changed plasma metabolite classes.

Metabolite Class	Metabolites Detected in the Class, *n*	Metabolites Significantly Affected, *n* (% of Class)	Metabolites Significantly Increased, *n* (% of Class)	Metabolites Significantly Decreased, *n* (% of Class)
**Amino acids**	193	33 (17.1)	19 (9.8)	14 (7.3)
**Carbohydrates**	23	3 (13.0)	1 (4.3)	2 (8.7)
**Cofactors and vitamins**	30	9 (30.0)	4 (13.3)	5 (16.7)
**Energy**	9	1 (11.1)	0 (0)	1 (11.1)
**Lipids**	369	154 (41.7)	37 (10.0)	117 (31.7)
**Nucleotides**	48	7 (14.6)	4 (8.3)	3 (6.3)
**Peptides**	29	6 (20.7)	4 (13.8)	2 (6.9)
**Xenobiotics**	91	9 (9.9)	6 (6.6)	3 (3.3)
**All metabolites**	801	222 (27.7)	75 (9.4)	147 (18.4)

Data are *n* or *n* (% of Class).

### Impact of dietary MCTs on energy lipids

#### Diacylglycerols

The univariate sum of diacylglycerols (DAGs) did not show a treatment effect (*P* = 0.111) (Table 3). However, the DAG class was different across treatments in a multivariate manner (MANOVA *P* < 0.001), with 5/9 (56%) of detected metabolites exhibiting a treatment effect by univariate LMM (range *P* = 4.4x10^−4^ to 0.029). One of the changed metabolites was decreased while the remaining four were increased (Table S6).

**Table 3:** Summary of detected and changed plasma metabolite classes relevant to energy metabolism.

Metabolite class	Control	Test	Delta test—control^a^	Class sum LMM *P*	MANOVA *P*	Metabolites detected in the Class, *n*	Metabolites significantly affected, *n* (% of Class)
**Diacylglycerols**	−2.08 ± 0.88	−0.21 ± 1.13	1.87	0.111	9.6E-04	9	5 (56)
**Saturated fatty acids**	0.51 ± 1.77	1.59 ± 1.53	1.07	0.345	0.432	25	2 (8)
**Unsaturated Fatty Acids**	−2.61 ± 3.75	−0.91 ± 3.26	1.70	0.507	N/A	34	3 (9)
**beta-Hydroxy Fatty Acids**	−0.38 ± 0.75	0.38 ± 0.72	0.76	0.355	0.436	11	2 (18)
**omega-Carboxy Fatty Acids**	−1.58 ± 0.99	−0.89 ± 0.93	0.68	0.219	4.3E-04	19	6 (32)
**Carnitine Adducts**	−5.06 ± 5.35	−6.63 ± 4.91	−1.57	0.398	N/A	58	19 (33)
**Endocannabinoids**	−1.02 ± 1.37	−0.67 ± 0.95	0.35	0.772	0.179	15	6 (40)
**Total**						171	43 (25)

All Log2 data. Data are mean ± standard error, *n*, or *n* (% of Class).

LMM, linear mixed model.

a Difference of the means.

#### Saturated fatty acids.

The univariate sum of saturated fatty acids (SFAs) did not show a treatment effect (*P* = 0.345). Additionally, the SFA class was not different across treatments in a multivariate manner (MANOVA *P* = 0.432), and only 2/25 (8%) of these metabolites were altered by food in a univariate LMM (range *P* = 0.028 to 0.048). The changed metabolites were increased after consumption of the test food as compared to the control food.

#### Unsaturated fatty acids

The univariate sum of unsaturated fatty acids (UFAs) did not show a treatment effect (*P* = 0.507) and MANOVA was not performed due to the large number of independent variables relative to the sample size. Only 3/34 (9%) of detected metabolites exhibited a food effect by univariate LMM (range *P* = 0.002–0.039). All of the changed metabolites were increased after consumption of the test food as compared to the control food.

#### Beta-hydroxy fatty acids

The univariate sum of beta-hydroxy fatty acids (βHFAs) did not show a treatment effect (*P* = 0.355). Additionally, the βHFA class was not different in a multivariate manner (MANOVA *P* = 0.436). However, 2/11 (18%) of these metabolites were significantly altered by food in a univariate LMM (range *P* = 0.020–0.040). Both of the changed metabolites, which included BHB, were increased after the cats consumed the C8 MCT-containing food.

#### Omega-carboxy fatty acids

The univariate sum of omega-carboxy fatty acids (ωCFAs) did not show a treatment effect (*P* = 0.219). However, the ωCFA class was different across treatments in a multivariate manner (MANOVA *P* = 4.3 × 10^−4^), with 6/19 (32%) of detected metabolites exhibiting a food effect by univariate LMM (range *P* = 2.6 × 10^−8^ to 0.036). Of the six altered ωCFAs, five were increased and one was decreased after consumption of the C8 MCT-enhanced food as compared to the control food.

#### Carnitine adducts

The univariate sum of carnitine adducts did not show a treatment effect (*P* = 0.398) and MANOVA was not performed due to the large number of independent variables relative to the sample size. However, there were 19/58 (33%) individual carnitine adduct metabolites that exhibited a treatment effect by univariate LMM (range *P* = 2.1 × 10^−8^ to 0.040). Of the 19 altered metabolites 8 were increased while the other 11 were decreased with the test food as compared to the control food.

#### Endocannabinoids

The univariate sum of endocannabinoids did not show a treatment effect (*P* = 0.772). Additionally, the endocannabinoid class was not different in a multivariate manner (MANOVA *P* = 0.179). However, 6/15 (40%) of these metabolites were significantly altered by food in a univariate LMM (range *P* = 7.1 × 10^−5^ to 0.047). Of the six altered metabolites three were increased while the other three were decreased after the cats consumed the C8 MCT-containing food.

### Impact of dietary MCTs on lipid immune modulators

#### Ceramides

The univariate sum of ceramides did not show a treatment effect (*P* = 0.294; Table 4). However, the ceramide class was different across treatments in a multivariate manner (MANOVA *P* = 0.009), with 4/8 (50%) of detected metabolites exhibiting a treatment effect by univariate LMM (range *P* = 6.2 × 10^−4^ to 0.038). Of the affected metabolites, one was increased and three were decreased after consumption of the test food as compared to the control food.

**Table 4:** Summary of detected and changed plasma metabolite classes relevant to lipid immune modulation.

Metabolite class	Control	Test	Delta test—control^a^	Class sum LMM *P*	MANOVA *P*	Metabolites detected in the class, *n*	Metabolites significantly affected, *n* (% of Class)
**Ceramides**	−0.06 ± 0.90	−0.89 ± 0.69	−0.82	0.294	0.009	8	4 (50)
**Dihydroceramides**	0.28 ± 0.23	−0.28 ± 0.28	−0.56	0.026	0.195	2	1 (50)
**Dihydrosphingomyelins**	0.83 ± 0.51	−1.12 ± 0.66	−1.95	3.6E-04	0.013	5	5 (100)
**Hexosylceramides**	0.15 ± 0.86	−2.96 ± 0.98	−3.11	0.001	0.203	8	7 (88)
**Lysophospholipids**	1.94 ± 1.79	−4.53 ± 1.67	−6.46	0.002	0.102	26	13 (50)
**Lysoplasmalogens**	0.36 ± 0.23	−0.79 ± 0.25	−1.15	5.4E-05	0.005	4	4 (100)
**Phosphatidylcholines**	0.50 ± 1.30	−1.81 ± 1.32	−2.31	0.040	6.6E-04	21	15 (71)
**Phosphatidylethanolamines**	−1.05 ± 0.89	0.22 ± 0.78	1.27	0.112	4.6E-04	8	5 (63)
**Phosphatidylinositols & Inositol Metabolism**	0.50 ± 0.73	−1.83 ± 0.81	−2.33	3.5E-05	4.0E-04	7	6 (86)
**Plasmalogens**	0.66 ± 0.41	−2.18 ± 0.66	−2.84	1.2E-04	0.003	9	8 (89)
**Sphingomyelins**	1.90 ± 2.24	−3.83 ± 2.24	−5.72	0.002	0.117	29	22 (76)
**Sphingosines**	0.42 ± 0.35	−0.07 ± 0.37	−0.50	0.238	0.018	4	2 (50)
**Sterols**	1.25 ± 0.74	−2.27 ± 0.67	−3.52	1.7E-06	0.063	8	5 (63)
**Total**						139	97 (70)

All Log2 data. Data are mean ± standard error, *n*, or *n* (% of Class).

LMM, linear mixed model.

a Difference of the means.

#### Dihydroceramides

The univariate sum of dihydroceramides showed a treatment effect (Log2 FC = −0.56; *P* = 0.026), but the dihydroceramide class was not different across treatments in a multivariate manner (MANOVA *P* = 0.195). However, 1/2 (50%) of these metabolites were altered by food in a univariate LMM (*P* = 0.004). The altered metabolite was decreased after the cats consumed the C8 MCT-containing food.

#### Dihydrosphingomyelins

The univariate sum of dihydrosphingomyelins showed a treatment effect (Log2 FC = −1.95; *P* = 3.6 × 10^−4^). The dihydrosphingomyelin class was also different across treatments in a multivariate manner (MANOVA *P* = 0.013), with all five metabolites in the class also exhibiting a treatment effect by univariate LMM (range *P* = 1.6 × 10^−7^ to 0.009). Four of these metabolites were decreased and one was increased after consumption of the test food as compared to the control food.

#### Hexosylceramides

The univariate sum of hexosylceramides (HCERs) showed a treatment effect (Log2 FC = −3.11; *P* = 6.4 × 10^−4^), but the HCER class was not different across treatments in a multivariate manner (MANOVA *P* = 0.203). However, 7/8 (88%) of these metabolites were altered by food in a univariate LMM (range *P* = 1.8 × 10^−5^ to 0.049). All of the altered metabolites in the class were decreased by the test food as compared to the control food.

#### Lysophospholipids

The univariate sum of lysophospholipids showed a treatment effect (Log2 FC = −6.46; *P* = 0.002), but the lysophospholipid class was not different across treatments in a multivariate manner (*P* = 0.102). However, 13/26 (50%) of these metabolites were altered by food in a univariate LMM (range *P* = 4.0 × 10^−7^ to 0.049). All of the altered metabolites in the class were decreased by the test food as compared to the control food. None of the glycerophosphatidic acid or glycerophosphoglycerol lysophospholipids were changed.

#### Lysoplasmalogens

The univariate sum of lysoplasmalogens showed a treatment effect (Log2 FC = −1.15; *P* = 5.4 × 10^−5^). Additionally, the lysoplasmalogen class was different across treatments in a multivariate manner (MANOVA *P* = 0.005), with all four metabolites in the class also exhibiting a food effect by univariate LMM (range *P* = 9.9 × 10^−6^ to 0.007). All of the metabolites in the class were decreased after consumption of the test food as compared to the control food.

#### Phosphatidylcholines

The univariate sum of phosphatidylcholines (PCs) showed a treatment effect (Log2 FC = −2.31; *P* = 0.040). Additionally, the PC class was different across treatments in a multivariate manner (MANOVA *P* = 6.6 × 10^−4^), with 15/21 (71%) of detected metabolites also exhibiting a treatment effect by univariate LMM (range *P* = 1.6 × 10^−8^ to 0.042). Of the 15 altered analytes, 12 were decreased after consumption of the C8 MCT-enhanced food as compared to the control food. The three metabolites that increased after eating the test food were docosahexaenoic acid (DHA)-containing PCs.

#### Phosphatidylethanolamines

The univariate sum of phosphatidylethanolamines (PEs) did not show a treatment effect (*P* = 0.112). However, the PE class was different across treatments in a multivariate manner (MANOVA *P* = 4.6 × 10^−4^), with 5/8 (63%) of detected metabolites also exhibiting a food effect by univariate LMM (range *P* = 3.8 × 10^−5^ to 0.023). Of the five altered metabolites, one was decreased while the other four were increased with the test food as opposed to the control food.

#### Phosphatidylinositols and inositol metabolism

The univariate sum of phosphatidylinositols (PIs) showed a treatment effect (Log2 FC = −2.33; *P* = 3.5 × 10^−5^). Additionally, the PI class was different across treatments in a multivariate manner (MANOVA *P* = 4.0 × 10^−4^), with 6/7 (86%) of detected metabolites also exhibiting a food effect by univariate LMM (range *P* = 9.8 × 10^−6^ to 0.035). While all six of the affected metabolites were decreased by the test food, palmitoyl-containing PIs seemed to be the drivers of the difference observed.

#### Plasmalogens

The univariate sum of plasmalogens showed a treatment effect (Log2 FC = −2.84; *P* = 1.2 × 10^−4^). Additionally, the plasmalogen class was different across treatments in a multivariate manner (MANOVA *P* = 0.003), with 8/9 (89%) of detected metabolites also exhibiting a food effect by univariate LMM (range *P* = 6.3 × 10^−6^ to 0.010). All of the altered metabolites in the class were decreased by the test food as compared to the control food.

#### Sphingomyelins

The sum of sphingomyelins showed a treatment effect by univariate LMM (Log2 FC = −5.72; *P* = 0.002), but the sphingomyelin class was not different across treatments in a multivariate manner (MANOVA *P* = 0.117). However, 22/29 (76%) of these metabolites were altered by food in a univariate LMM (range *P* = 7.1 × 10^−6^ to 0.047). All of the altered metabolites in the class were decreased by the test food as compared to the control food.

#### Sphingosines

The univariate sum of sphingosines did not show a treatment effect (*P* = 0.238). However, the sphingosine class was different across treatments in a multivariate manner (MANOVA *P* = 0.018), with 2/4 (50%) of detected metabolites also exhibiting a food effect by univariate LMM (range *P* = 0.020–0.045). One of the changed metabolites was increased while the other was decreased by the test food as compared to the control food.

#### Sterols

The univariate sum of sterols showed a treatment effect (Log2 FC = −3.52; *P* = 1.7 × 10^−6^), but the sterol class was not different across treatments in a multivariate manner (*P* = 0.063). However, 5/8 (63%) of these metabolites were altered by food in a univariate LMM (range *P* = 3.9 × 10^−9^ to 0.001). All of the altered metabolites in the class were decreased by the test food as compared to the control food.

### Impact of dietary MCTs on intermediates of carbohydrate and amino acid metabolism

The metabolites of pantothenate (Log2 FC = 0.21; *P* = 0.001), glycerate (Log2 FC = 0.17; *P* = 0.003), citrate (Log2 FC = −0.08; *P* = 0.022), maltose (Log2 FC = −0.60; *P* = 0.031), trans-4-hydroxyproline (Log2 FC = −0.11; *P* = 0.004), and pyrraline (Log2 FC = −0.37; *P* = 0.009) exhibited a treatment effect by univariate LMM.

## Discussion

A recent review has shown numerous health benefits of consuming dietary MCTs including but not limited to increased energy metabolism, improved immune function and cognition, and ameliorated atherosclerosis in the context of human health and disease (Roopashree et al. 2021). The current study demonstrated that feeding a food containing 8% C8 MCTs increased available energy in the form of BHB in both serum and plasma in cats with CKD. A human study suggests the ketone BHB is widely used throughout the body as an energy source for production of ATP, and is actually the preferred energy source of the kidneys (Cuenoud et al. 2020).

A study in humans with multiple sclerosis showed that a ketogenic diet increased BHB levels and lean mass (Benlloch et al. 2019). Another study in healthy humans revealed that BHB has potent anticatabolic effects during catabolic conditions such as CKD and other inflammatory disorders (Thomsen et al. 2018). Urine creatinine concentration can also be a measure of muscle mass or turnover (Barzilay et al. 2021). Urine total protein and urine creatinine concentration, common markers of kidney health, were decreased in the current study after consumption of the test food. Reduced urine protein is a sign of improved filtration in the glomerulus and thus indicates increased kidney health. Decreased concentration of urine creatinine can imply a reduction in the kidneys’ ability to concentrate urine. However, the current study did not see a change in the urine protein: creatinine ratio or uSG by food, which suggests the kidneys’ concentrating ability has not changed after the addition of C8 MCT.

Cats fed the test food showed upregulated metabolites pantothenate, a precursor to coenzyme A (CoA) synthesis, and glycerate (intermediate in glycolysis). Citrate (intermediate in tricarboxylic acid (TCA) cycle and inhibitor of the glycolytic enzyme phosphofructokinase) and maltose (intermediate in glycogen metabolism and glycolysis) were downregulated. Because pantothenate is a precursor to CoA, and CoA is required for the TCA cycle, an increase in pantothenate may suggest an enhancement of TCA cycle activity (Barritt et al. 2024). However, CoA is also involved in FAO and many other cellular processes such as the regulation of DNA repair and gene expression; thus, upregulated pantothenate may result from various factors (Barritt et al. 2024). Glycerate is involved in glycolysis and pyruvate metabolism and can be utilized in the synthesis of serine through various pathways involving pyruvate. Serine has been described as a major contributor of one-carbon units for protein and nucleic acid methylation and is involved in the synthesis of many metabolic intermediates, making it an essential component of cellular metabolism (Kalhan and Hanson 2012). Further, studies done in humans and rats have shown the primary site of serine synthesis during fasting to be the kidneys (Kalhan and Hanson 2012). Results from the current study did not indicate a change in serine or pyruvate after consumption of the test food; however, an increase in glycerate may indirectly suggest increased kidney function or enhancement of metabolic pathways which utilize serine or pyruvate. Betaine is another contributor of one-carbon units and is known to have anti-inflammatory and antioxidant properties (Ephraim and Jewell 2023). This metabolite showed a trend toward upregulation in the current study, further indicating a possible benefit in the treatment of CKD.

Citrate functions as an intermediate in the TCA cycle and as an allosteric inhibitor of phosphofructokinase, which converts fructose 6-phosphate to fructose 1,6-bisphosphate in the third step of glycolysis (Sivadas et al. 2023). A study on nitric oxide production in murine macrophages during polarization has described high levels of citrate to be a result of defective TCA cycle activity and as a contributor for inflammatory lipid mediator synthesis (Palmieri et al. 2020). Therefore, downregulated citrate indicates alterations to the energy pathways of glycolysis and the TCA cycle, which are dependent on a number of factors. Maltose is a disaccharide produced from the partial breakdown of starch or glycogen and is composed of two glucose molecules, making it an important intermediate in glycogen metabolism and glycolysis. While decreased maltose could merely indicate reduced dietary starch intake or a state of fasting, it could also imply that maltose is being efficiently metabolized into glucose or glycogen or that less glycogen is being broken down. Overall, the alteration in these metabolites indicates that there is a shift from carbohydrate energy production (glycolysis) to fat energy production (FAO) in cats with CKD while consuming a food enriched with C8 MCTs.

FAO is a major source of energy for highly metabolic cells, including healthy renal tubule epithelial cells, in human CKD tissue and mouse models of kidney fibrosis (Kang et al. 2015). Additionally, a review on human mitochondrial FAO reports that this pathway stimulates ketone body synthesis in the liver (Houten et al. 2016). Diacylglycerols have been described to be an intermediate in the synthesis of more complex lipids, a source of free fatty acids, and a product of dihydroxyacetone-3-phosphate (a glycolytic intermediate; Carrasco and Merida 2007). Among the energy metabolite classes, 27/171 (15.8%) individual metabolites were upregulated and 16/171 (9.4%) metabolites were downregulated after cats consumed the C8 MCT enriched food, indicating more substrates were available for FAO and further suggesting a shift away from glycolysis and towards FAO. The absence of an observed increase in circulating saturated fatty acids in the current study, despite a higher dietary content, is likely because of their rapid oxidation. Given that blood samples were collected 16 hr after the last meal, the circulating metabolites measured do not reflect acute postprandial lipid profiles. Instead, they indicate longer-term metabolic effects. This is consistent with observations in healthy cats, where mean serum and plasma triglyceride levels typically peak 3–4 hr after a fat-containing meal and return to baseline 7–8 hr post-feeding (Ginzinger et al. 1999; Kluger et al. 2009). Therefore, the saturated fatty acid levels measured after a 16-hr fast reflect the longer-term metabolic status rather than an immediate response to the recent meal.

Endocannabinoids regulate diverse biological functions including neurological development and function, energy metabolism and homeostasis, and immune function (Silvestri and Di Marzo 2013; Turcotte et al. 2015; Almeida et al. 2022; Arceri et al. 2023). In the present study, all upregulated endocannabinoids were taurine-conjugated fatty acids, consistent with human data showing that taurine supplementation can enhance mitochondrial activity and lipid metabolism (de Carvalho et al. 2020, 2021). Thus, the observed changes in taurine-conjugated endocannabinoids are compatible with a shift toward increased lipid-derived energy production. Although, the primary functions of the endocannabinoid system are traditionally associated with cognition and neuromodulation, numerous studies have demonstrated that the broader endocannabinoidome plays a notable role in renal pathology, homeostasis, and function (Turcotte et al. 2015; Chua et al. 2019; Peritore et al. 2019; Arceri et al. 2023). In this context, all three of the endocannabinoid metabolites that were downregulated after cats consumed the MCT-enriched food belong to a family of molecules called ALIAmides, which are a group of naturally occurring bioactive acyl ethanolamides that exhibit anti-inflammatory effects (Peritore et al. 2019). Moreover, two of these downregulated ALIAmides have been shown to act via binding to peroxisome proliferator-activated receptor-alpha (PPAR-α, regulator of lipid metabolism), and transient receptor potential vanilloid 1 (involved in pain and immune modulation; Ezzili et al. 2010; Silvestri and Di Marzo 2013; Peritore et al. 2019; Almeida et al. 2022). The intricate process of inflammation and its resolution is modified by several mechanisms and involves a complex interplay of immune and endocrine molecules. Lipid mediators and immune cell function, including production of pro-inflammatory cytokines and chemokines, is critical to initiating and regulating the inflammatory response. While a decrease in ALIAmides may induce a pro-inflammatory state, determining if the anti-inflammatory properties of taurine-conjugated endocannabinoids are sufficient to counteract this potential effect is a subject for future research.

According to a review on human health, eicosanoids have various roles as mediators and regulators of inflammation and the ω-6 PUFA arachidonic acid (AA) is known to increase eicosanoid formation under stressed conditions; therefore, eicosanoids and AA have been linked to inflammatory diseases (Djuricic and Calder 2021). On the other hand, the ω-3 PUFAs eicosapentaenoic acid (EPA) and DHA have been shown to have anti-inflammatory effects and decrease production of AA-derived eicosanoids (Djuricic and Calder 2021). However, studies have shown that ω-3 and ω-6 PUFAs such as AA, DHA, and EPA are all employed in the biosynthesis of endogenous specialized pro-resolving lipid mediators, which aid in the resolution of inflammation (Huwiler and Pfeilschifter 2009; Brennan et al. 2021; Djuricic and Calder 2021; Giardini et al. 2024). While the individual phospholipid metabolites of AA, DHA, and EPA were not altered in the current study, some products of DHA (DHA-containing PCs and PEs) were upregulated. In addition, among the seven detected AA-containing PCs, PEs, and PIs, three of the four metabolites that were altered were downregulated. The study’s findings demonstrate that consuming a diet enriched with C8 MCTs can reduce inflammation in cats with CKD, as evidenced by the upregulation of DHA-containing phospholipids and the downregulation of AA-containing phospholipids. Future research should also consider measuring downstream products of these phospholipid metabolites, such as lipoxins, resolvins, and protectins, which were not assessed in the current study.

In a review of both human and mouse anti-inflammatory therapies, ceramides were described as being pro-inflammatory due to their ability to stimulate phospholipases A_2_ (PLA_2_), contributing to AA release (Huwiler and Pfeilschifter 2009). A downregulation in the ceramide and dihydroceramide (a precursor to ceramide) metabolite classes was seen in the current study. There was also a decrease in the sphingosine class; however, one individual metabolite from this class, sphingosine 1-phosphate (S1P), was upregulated. S1P has been described as being anti-inflammatory in a number of previous studies and is proposed to act as a counter-molecule of ceramide, making S1P and ceramide regulators of inflammatory processes (Huwiler and Pfeilschifter 2009). C-reactive protein (CRP) and serum amyloid A are markers of systemic inflammation and were shown to be positively correlated with a number of sphingomyelins and dihydrosphingomyelins and negatively correlated with a number of lysophospholipids in a human study exploring the associations between plasma metabolites and inflammatory biomarkers (Gao et al. 2021). The current study observed a decrease in all three of these metabolite classes when cats ate the C8 MCT-enriched food, suggesting the presence of both pro-inflammatory and anti-inflammatory effects. Statins, drugs used to lower blood cholesterol levels, have been shown to decrease CRP levels in humans with CKD (Kadatane et al. 2023). Additionally, the accumulation of cholesterol has been shown to induce expression of the NLRP3 inflammasome components in an immortalized mouse cortical collecting duct cell model (Kong et al. 2020). Consistent with these mechanisms, decreases in the individual cholesterol metabolite and circulating blood cholesterol were detected in the current study, suggesting a reduction in the inflammatory signal. Taken together, the data demonstrates that consumption of a food enriched with C8 MCTs can reduce inflammation through reduced cholesterol levels.

Persistent oxidative stress, as demonstrated in both human studies and animal models of CKD, amplifies cytokine overproduction and inflammation, causing damage to renal tubular cells and glomeruli and thereby promoting further progression of renal disease (Gyuraszova et al. 2019; Rapa et al. 2019). Previous studies have shown advanced glycation end products (AGEs), such as pyrraline, increase oxidative stress and inflammatory states through various mechanisms in humans (Twarda-Clapa et al. 2022). Dietary AGEs have also been shown to influence urinary excretion of AGEs in cats (Palaseweenun et al. 2021); however, to the authors’ knowledge, no studies have evaluated the biological or clinical relevance of AGEs in cats. Given that inflammation is a known factor in the progression of CKD due to its ability to provoke renal fibrosis (Lawson et al. 2015), elucidating the role of AGEs on oxidative stress and inflammation in cats should be a focus of future research. Trans-4-hydroxyproline is an amino acid that is a key component of the protein collagen, which is the most abundant protein in the extracellular matrix (Hall et al. 2022), and collagen degradation is a sign of extracellular matrix remodeling and renal fibrosis (Ephraim and Jewell 2023). The current study saw a reduction in pyrraline and trans-4-hydroxyproline, which together suggest a decrease in oxidative stress, inflammation, and fibrosis.

Some limitations of the current study include the relatively short washout period (seven days), the brief overall study duration, and the small sample size. Additionally, metabolomics data was reported with the lowest detectable values when the true value was below the detection limit, which could have diminished our ability to detect true effects. Renal values such as blood creatinine and SDMA did not change over the course of the study; however, the findings suggest conducting a longer feeding study may positively influence renal markers—a possibility that warrants future evaluation. In summary, these results indicate feeding C8 MCTs to cats with CKD enhances energy availability from ketones and reduces lipids associated with inflammation. Therefore, feeding C8 MCTs appears to be beneficial in the management of CKD in cats by providing more available energy and reducing markers of inflammation.

## Supplementary Material

skag193_Supplementary_Data

## Data Availability

The data that support the findings of this study are available from the corresponding author upon request.

## Abbreviations

AA: arachidonic acid
AGE: advanced glycation end product
Alb/Glob: albumin/globulin
ALP: alkaline phosphatase
ATP: adenosine triphosphate
BHB: beta-hydroxybutyrate
βHFA: beta-hydroxy fatty acid
CBC: complete blood count
CKD: chronic kidney disease
CoA: coenzyme A
CPT1: carnitine palmitoyl transferase 1
CRP: C-reactive protein
DAG: diacylglycerol
DHA: docosahexaenoic acid
EPA: eicosapentaenoic acid
FAO: fatty acid beta-oxidation
FC: fold change
GPA: glycerophosphatidic acid
GPG: glycerophosphoglycerol
HCER: hexosylceramide
IRF: immature reticulocyte fraction
IRIS: International Renal Interest Society
LMM: linear mixed model
MANOVA: multivariate analysis of variance
MCFA: medium-chain fatty acid
MCT: medium-chain triglyceride
NLRP3: Nod-like receptor family pyrin domain containing 3
OXPHOS: oxidative phosphorylation
ωCFA: omega-carboxy fatty acid
PC: phosphatidylcholine
PE: phosphatidylethanolamine
PI: phosphatidylinositol
PLA2: phospholipases A2
PPAR-α: peroxisome proliferator-activated receptor-alpha
PUFA: polyunsaturated fatty acid
RDW: red blood cell distribution width
S1P: sphingosine 1-phosphate
SAA: serum amyloid A
SDMA: symmetric dimethylarginine
SFA: saturated fatty acid
SPM: specialized pro-resolving lipid mediator
TCA: tricarboxylic acid
TRPV1: transient receptor potential vanilloid 1
UFA: unsaturated fatty acid
UPC: urine protein: creatinine ratio
uSG: urine specific gravity
